# Generation of Blood Vascular Endothelial-Neural 3D Organoids by Serial Induction of Differentiation on Human iPSC-Derived Embryoid Bodies

**DOI:** 10.3390/cells15131192

**Published:** 2026-06-30

**Authors:** Tongguang Wang, Anna Bagnell, Valerie McDonald, Benjamin D. Gastfriend, Joseph P. Steiner, Abdel G. Elkahloun, Kory Johnson, Rebekah G. Langston, Mark R. Cookson, Avindra Nath

**Affiliations:** 1 Translational Neuroscience Center, National Institute of Neurological Disorders and Stroke, National Institutes of Health, Bethesda, MD 20892, USAjoe.steiner@nih.gov (J.P.S.); 2 Cancer Genetics Branch, National Human Genome Research Institute, National Institutes of Health, Bethesda, MD 20892, USA; 3 Bioinformatics Section, National Institute of Neurological Disorders and Stroke, National Institutes of Health, Bethesda, MD 20892, USA; 4 Cell Biology and Gene Expression Section, National Institute on Aging, National Institutes of Health, Bethesda, MD 20892, USA; 5 Section of Infections of the Nervous System, National Institute of Neurological Disorders and Stroke, National Institutes of Health, Bethesda, MD 20892, USA

**Keywords:** brain organoid, 3D organoid, vascular endothelial cells, iPSC, neural induction

## Abstract

**Highlights:**

**What are the main findings?**

- We reconstitute a 3D organoid model by serially inducing endothelial and neural differentiation to mimic the in vivo development of neural and endothelial cells.
- The resulting 3D organoid consists of a variety of neurons, glial and endothelial cells with vascular-like structures, comparable to the human brain.

**What is the implication of the main findings?**

- This EC-neural organoid model provides a useful tool to study the interactions between vascular endothelial cells and neural cells in early brain development.
- These EC-neural organoids can potentially be used to model neural infectious disorders where endothelial cells are targets and used as mediators of neuronal damage.

**Abstract:**

The 3D brain organoids have been widely used as a tool to study human brain development and disorders. Although angiogenesis and blood vascular endothelial cells play important roles in brain development and pathogenesis in neurological disorders, most 3D brain organoids lack inherent endothelial cells and need either the addition of differentiated endothelial cells or to be transplanted to animals to reconstitute such vascular structures. However, these approaches could miss the developmental interactions between angiogenesis and neurogenesis in the human brain. To reconstitute a 3D organoid mimicking the in vivo development of neural and vascular endothelial cells, we cultured iPSC-derived embryoid bodies and sequentially applied endothelial and neuronal induction media along with Matrigel embedding. The resulting 3D organoid consists of both neural cells and endothelial cells with vascular-like structures, as determined by immunostaining. With scRNA-Seq analysis, the organoid was confirmed to contain neural cell types similar to human brains, including a variety of excitatory and inhibitory neurons and glia. Furthermore, when compared with conventionally generated cerebral organoids without endothelial cells using RNA-Seq analysis, the vascular endothelial-containing neural organoids (EC-neural organoids) showed different gene profiles and favored angiogenesis and vasculogenesis. Of the differentially expressed genes, KRBA2 expression was found to be higher in neural cells and its inhibition by siRNA treatment resulted in decreased transcriptions of a variety of genes specific to neuronal differentiation but not genes specific to pluripotent stem cells such as OCT4. The EC-neural organoids also expressed receptors to SARS-CoV-2 at levels similar to human brains. This 3D EC-neural model provides a useful tool to study the interactions between vascular endothelial cells and neural cells in brain development and potentially for the study of neural infectious disorders where vascular endothelial cells are targets for infection and mediators for neural damage.

## 1. Introduction

Organoids derived from human stem cells have been developed in recent years as an alternative in vitro model for animal and human subject studies. With relatively fewer ethical concerns and wide feasibility, human organoids constitute multiple cell types and tissue structures similar to the corresponding human organs. Set to partly substitute animal models, they represent the future of human disease modeling and have the potential to play a pivotal role in the study of disease pathogenesis, drug development and guidance of individualized therapies. There are reported successes in the treatment of cystic fibrosis [1] and some tumors. As such, studies using 3D brain organoids derived from human induced pluripotent stem cells (iPSCs) have also prevailed in the field of neuroscience, modeling neural infectious diseases including ZIKA, SARS-CoV-2 and neurodegenerative diseases including amyotrophic lateral sclerosis (ALS) and Alzheimer’s disease. However, conventional 3D neural organoids lack certain brain cell types, such as microglia and endothelial cells, as they were derived from other germ layers than neural cells. Evidence suggests that the pathogenesis of neurological disorders may not be limited to neurons or the central nervous system but the result of complicated interactions involving multiple organs, even from imbalances in the gut microbiota [2]. As for modeling neural infectious diseases, the neural organoids without endothelial cells are inadequate because infectious agents usually target blood vascular endothelial cells first before infecting the neural cells. To reconstitute vascular endothelial cells or blood vessels into the brain organoids, separately differentiated endothelial cells have been added to neural spheres [3]. Alternatively, the neural organoids can be transplanted into an animal brain to get vascularized [4]. These approaches either miss the initial interactions between endothelial cells and neural cells during brain development or involve complicated techniques such as animal operation, which undermine the benefits of using in vitro 3D brain organoids.

The interactions between neural and vascular endothelial cells play important roles in the development of the neural system and its normal function. Besides serving as conduits for the delivery of oxygen and nutrients, perfusion-independent roles of organotypic endothelium play during brain development and regeneration through releasing specific paracrine growth factors called angiocrine growth factors or angiokines [5]. For example, we have reported that VEGF can function as a regulator for oligodendrocyte proliferation and differentiation [6]. On the other hand, it is well known that endothelial cells exhibit distinct functional and molecular heterogeneity depending on the types of vessels, organs and especially age. Although not well studied, it is conceived that endothelial cells and hematopoietic cells develop in parallel and the latter arise from specialized endothelial cells [7]. There are endothelial cells that exist as stem/progenitor like cells which continuously proliferate and are ready for angiogenesis even in adults. It is known that, different from neural cells originating from exoderm, blood vascular endothelial cells are from mesoderm whose specification is initiated by BMP4 and bFGF [8]. Expression of VEGFR2 on angioblasts is necessary for allowing their communication with the binding ligand VEGF-A, which further facilitates vascular endothelial cell development in a highly controlled manner. Thus, it is possible to derive vascular endothelial cells by mimicking the in vivo process alongside neural differentiation.

To address these issues, we developed a 3D neural organoid model by inducing vascular endothelial cell differentiation with serial applications of BMP4, bFGF and VEGF onto iPSC-derived embryoid bodies, followed by neuronal differentiation and maturation media. The resulting organoids contain vascular endothelial cells and varieties of neural cells similar to the human brain. By comparing them with traditional neural organoids, we found certain differentially expressed genes that may play unique roles in neural development. In addition, we screened for SARS-CoV-2 receptors on the endothelial-containing organoids (EC-neural organoids) and found a similar distribution as in human brains. These results indicate the EC-neural organoids can be used as a model to study human neural development and potentially neural infections such as SARS-CoV-2.

## 2. Materials and Methods

### 2.1. Reagents and Supplies

Culture media and components were purchased from Invitrogen (Carlsbad, CA, USA); growth factors and cytokines were purchased from PeproTech (Rocky Hill, NJ, USA); and chemicals were purchased from Sigma (St. Louis, MO, USA) if not otherwise specified. Details of the reagents and resources were provided in the Supplementary Materials, Table S1.

### 2.2. Cell Cultures

Peripheral blood mononuclear cells (PBMCs) were obtained from participants enrolled in the Baltimore Longitudinal Study of Aging (BLSA), a longitudinal cohort study conducted by the National Institute on Aging (NIA), NIH. Written informed consent was obtained from all participants, and all study procedures were approved by the appropriate NIH Institutional Review Board and conducted in accordance with the Declaration of Helsinki. Human induced pluripotent stem cell (iPSC) lines 505, 506 and 516 were generated from PBMCs using the Sendai virus method by the National Heart, Lung and Blood Institute iPSC core facility at NIH as published previously [9]. The iPSCs were cultured in E8 Flex medium on Matrigel-coated plates in a 5% CO_2_ incubator at 37 °C and sub-cultured using ethylene diamine tetra acetic acid (EDTA), following published protocols [10].

### 2.3. Culture 3D EC-Neural Organoids

Embryoid bodies were derived from iPSCs using AggreWell^TM^ plates (Stemcell Technologies, Vancouver, BC, Canada) according to the manufacturer’s instructions. Briefly, iPSCs were dissociated into single cells by treatment with Accutase dissociation buffer (Invitrogen) and counted. One million cells per well were seeded onto a low-attachment 24-well AggreWell plate in E8 Flex medium with ROCK inhibitor Y-27632 (10 μM). After 24 h, embryoid bodies were formed and transferred to new 24-well plates pretreated with anti-adherence rinsing solution (Stemcell Technologies) at ~10 spheres per well in 1 mL of E8 Flex medium.

To make 3D EC-organoids, 48 h after being transferred to a new plate, the embryoid bodies were coated by adding an ice-cold Matrigel drop wisely (25 μL/500 μL medium) into the medium and then incubated in a 5% CO_2_ incubator at 37 °C for 4 h. The endothelial cell induction process was done following modifications to a protocol used in 2D endothelial induction from iPSCs [11]. Briefly, 500 μL of medium consisting of DMEM/F12, 1X B27 supplements and Activin A (125 ng/mL) was added on top of the spheres, followed by incubation for 24 h. Then, 500 μL of the medium was replaced with freshly made E8 Flex consisting of 10 ng/mL of BMP4, followed by another 25 μL of ice-cold Matrigel overlay and incubation for 72 h. The medium was then changed to DMEM/F12 consisting of 1× B27 supplement, 1 mM of 8-bromo-cAMP, 100 ng/mL of vascular endothelial growth factor (VEGF) and antibiotics for 72 h. After that, half the medium was changed to DMEM/F12 medium consisting of 1× N2 supplement, 1× B27 supplement, 50 ng/mL of VEGF and antibiotic-antimycotic and this was done every other day to facilitate neural differentiation and maintain the growth of the organoids. The organoids were transferred to a 6-well plate pretreated with anti-adherence rinsing solution after 2 weeks in culture to avoid the overcrowding of cells.

The 3D cerebral organoids without endothelial cell induction were also derived from iPSCs using the Kit from Stem Cell Technologies with modifications. Following embryoid body formation using the Aggrewells, the neural induction and expansion were done as instructed and at day 5, the Matrigel was used to coat the spheroids drop wisely. The organoids were then transferred to maturation media for further neuronal maturation.

### 2.4. Organoid Clearance and Immunocytochemistry

For immunostaining, a clearing process was performed on the organoids using a Kit purchased from Visikol, following the manufacturer’s instructions with optimization. Briefly, the organoids were fixed in 4% paraformaldehyde (PFA) for 24 h in a cold room and then washed 2 times, 1 h each, with phosphate-buffered saline pH 7.4 (DPBS) to remove the PFA residue. Then, dehydration/permeabilization was done at 4 °C with gentle shaking for 10 min in each of the following buffers subsequently: 50% methanol in PBS, 80% methanol in water, 100% methanol, 20% DMSO in methanol, 80% methanol in water, 50% methanol in PBS, 100% PBS and PBS with 0.2% Triton X-100. After incubation for 30 min with penetration buffer (0.2% Triton X-100, 0.3 M glycine and 20% DMSO), the organoids were blocked with blocking buffer (DPBS with 0.2% Triton X-100, 6% donkey serum and 10% DMSO) for 30 min. The organoids were then incubated with the primary antibodies (rabbit anti-MAP2, Abcam, Waltham, MA, USA; rabbit anti-NG2, Abcam; rabbit anti-PDGFR-beat and mouse monoclonal anti-CD31, Thermo Fisher Scientific, Waltham, MA, USA) at 1:100 in DPBS with 0.2% Tween 20, 3% donkey serum and 5% DMSO for 1 h at 37 °C and then followed by 4 °C overnight. After that, the organoids were washed five times in DPBS with 0.2% Tween 20 and 100 μg/mL heparin for 10 min each time. The organoids were then incubated with corresponding secondary antibodies (1:100, anti-mouse Alexa Fluor 488; anti-rabbit Alexa Fluor 594: Thermo Fisher Scientific) at 37 °C for 1 h, followed by 1 h of 2-(4-amidinophenyl)-1H-indole-6-carboxamidine (DAPI) nuclear staining at room temperature. Then the organoids were washed for at least 10 times at 37 °C with shaking, 10 min each. When ready for imaging, the buffer was replaced with 200 mL of HISTO M clearing solution (Visikol, Hampton, NJ, USA). Images were acquired using a Zeiss LSM 510 META multiphoton confocal system (Carl Zeiss, Oberkochen, Germany) or ImageXpress Micro confocal microscope (Molecular Devices, San Jose, CA, USA).

### 2.5. Single-Cell RNA-Seq Analysis

Organoids were dissociated to single cells by treatment with Accutase for 20 min with shaking. Single-cell suspension quality, number and viability were assessed with a dual fluorescence cell counter, Luna-FL (Logos Biosystems, Gunpo-si, Republic of Korea). An amount of 8000–10,000 cells was targeted from each normal cell suspension. The cells were washed twice with PBS + 0.04% bovine serum albumin and resuspended at about 1000 cells per microliter. A 10× Genomics’ Chromium instrument and Single-Cell 3′ Reagent kit (V3 and 3.1) were used to prepare the individually barcoded single-cell RNA-sequencing libraries following the manufacturer’s protocol. Quality of the libraries was assessed by the Bioanalyzer traces (Agilent BioAnalyzer High Sensitivity Kit, Agilent Technologies, Santa Clara, CA, USA) and quantified by the Qubit system. Sequencing was done on the Illumina NextSeq machine, using the 150-cycle High-Output kit with 28 bp read 1, 8 bp sample index, and 91 bp read 2. Following sequencing, the bcl files were demultiplexed into FASTQ files, aligned to the human transcriptome GRCh38 and single-cell 3′ gene counting was performed by the standard 10× Genomics’s CellRanger mkfastq software (V3.0.2). The gene expression in single cells was visualized using 10× Genomics Loupe Browser 4.0.0. Pseudotime of differentiation trajectories was annotated by using Partek Flow scRNA-Seq analysis tools. (https://partekflow.cit.nih.gov/flow, accessed on 13 October 2021). Single-cell RNA-seq datasets generated from 3 EC-neural organoid samples were integrated with 21 single-nuclei RNA-seq datasets generated from post-mortem human frontal cortex. RNAseq data analyses of the human frontal cortex data have been previously published [12,13]. A total of 186,095 FCTx nuclei and 9061 organoid cells were analyzed after filtering. The datasets were normalized using SCTransform [14] and integrated by pairwise comparison of anchor gene expression [15] within the Seurat package [16] in R. Clustering of the integrated dataset was performed using a shared nearest neighbor approach and clusters were visualized using Uniform Manifold Approximation and Projection (UMAP). Clusters were manually assigned cell type identities based on differential expression of established cell type marker genes. For neuronal subtypes, the broad neuron classification categories identified in the Allen Brain Atlas Transcriptome Explorer of the “Human-M1-10x” dataset (e.g., PVALB+ inhibitory neurons = InN.PVALB) were used when appropriate [17]. There were two neuronal populations without a clear correlate in the Allen Brain Atlas data: an excitatory neuron cluster (ExN) characterized by differentially high expression of LINC00507 and an inhibitory neuron cluster (InN) with differentially high expression of PAX6. Non-neuronal cell populations including astrocytes (ASTs), oligodendrocyte precursor cells (OPCs), microglia (MGL), vascular leptomeningeal cells (VLMCs), endothelial cells (ECs) and oligodendrocytes (ODCs) were identified based on differential expression of known marker genes of these human brain cell types [18,19,20]. There was one cell cluster with markers of multiple cell types, labeled “mixed”. A Pearson correlation table measuring overall correlation of all of those genes (Figure S7), and tables showing the number of organoid cells assigned to each named cluster were provided in the Supplemental Materials.

### 2.6. Neural Stem Cell Differentiation and Small Inhibitory RNA (siRNA) Transfection

Neural stem cells (NSCs) were derived directly from cord blood CD34 cells (CD34-iNSC) [21] or differentiated from iPSCs (NSC 507 and NCRM) as reported in our previous publication [22]. For differentiation from iPSCs, iPSCs were seeded on a Matrigel-coated 6-well plate in E8 medium. When ready for induction, the E8 medium was replaced with PSC neural induction media (Gibco) for 7 days. The resulting NSCs were characterized by nestin immunostaining and were further differentiated to neurons when subcultured on Matrigel-coated plates and incubated with neuronal differentiation medium (DMEM/F12 containing 1×N2 supplement, 1×B27 supplement, 300 ng/mL cyclic adenosine monophosphate (cAMP, Sigma, Kawasaki-shi, Japan) and 0.2 mM vitamin C (Sigma), 10 ng/mL brain-derived neurotrophic factor (BDNF) and 10 ng/mL glial-derived neurotrophic factor (GDNF) for 14 days. The resulting neurons were confirmed by immunostaining for β-III-tubulin. Premade TriFECTa RNAi kit targeting human KRBA2 (hs.Ri.KRBA2.13) and IGFBP6 (hs.Ri.IGFBP6.13.1, hs.Ri.IGFBP6.13.2 and hs.Ri.IGFBP6.13.3), as well as the non-specific control RNAi, were purchased from IDT. For siRNA transfection, NSCs were cultured for 24 h and reached 80% confluence. TransIT-siQUEST transfection reagent (Mirus Bio, Madison, WI, USA) was mixed with RNAi (30 nM final concentration). After 15 min, the mixture was applied to the NSC cultures. The cells were then lysed for RNA purification after 48 h.

### 2.7. Real-Time Polymerase Chain Reaction (RT-PCR) Assay

RNA was extracted and purified using a total RNA PLUS purification kit (Norgen, Thorold, ON, Canada) following the manufacturer’s instructions. First-strand cDNA was synthesized using QuantiTect Reverse Transcription kit (Qiagen, Venlo, The Netherlands) following the manufacturer’s instructions. qPCR was conducted using FastStart Universal SYBR Green Master (ROX) kit from Roche and ViiA7 real-time PCR system (Life Technologies) according to the manufacturer’s instructions. Herv-K Env, Nestin, MAP2 and beta-2-microglobulin (B2M) primers were manufactured by Invitrogen as published before [9]. KRBA2 and IGFBP6 primers were purchased from IDT. The sequences of the primers were reported in the Supplemental Table S1. Delta Ct Value (ΔCt) was calculated using the accompanying software with the ViiA7 real-time PCR machine. ΔΔCt method was used for the differential gene expression analysis.

### 2.8. Analysis of Published Literature

Allen Atlas’s microarray database (2010 Allen Institute for Brain Science; Allen Human Brain Atlas. Available from: http://human.brain-map.org, accessed on 18 July 2010) [23] was used to compare the gene expression profiles between brain organoids and normal human brains and FURIN and BSG in human brains. The cell specificity of gene expression was checked using the cell transcriptomic RNA-Seq data tool in the Allen brain database (Allen Cell Types Database 2015, http://celltypes.brain-map.org/rnaseq/human_m1_10x, accessed on 17 July 2010). These results are shown as heatmaps. The levels of ACE2 and TMPRSS2 were also examined in a developing brain [24] using the UCSC cell browser v0.7.11 (http://genome.ucsc.edu/) [25].

### 2.9. Experimental Design and Statistical Analysis

For conceptual proof, three batches of brain organoids from different iPSC lines were derived independently and immunostained for the neuronal and endothelial cell markers. Four brain organoids were used to study the levels of SARS-CoV-2 infection-associated genes using scRNA-Seq analysis. Three were also used to assess their distributions among brain cell types. For statistical analysis, at least three independent experiments were performed. Mean and standard error (SE) were calculated for each treatment group. For RT-PCR results, ∆Ct or ∆∆Ct values were calculated. For comparison of gene expression, the levels of gene expression were presented as the fold of the highest expression treatment group. As a result, a one-sample *t* test was performed against the hypothetical value 1. Statistical analysis was performed using Prism, version 3.0. For the RNA-seq analysis, comparison between paired EC-neural organoids (E) and conventional neural organoids (N) derived from six iPSC lines was performed and genes were selected to be differentially expressed between N and E if they had an absolute linear fold change ≥ 2X and an uncorrected *p*-value < 0.01 by the paired t-test. Benjamini–Hochberg (BH) False Discovery Rate (FDR) method was used to confirm the significant difference.

## 3. Results

### 3.1. EC-Neural Organoids Were Generated with Neural and Vascular Endothelial Cells

**Figure 1:**
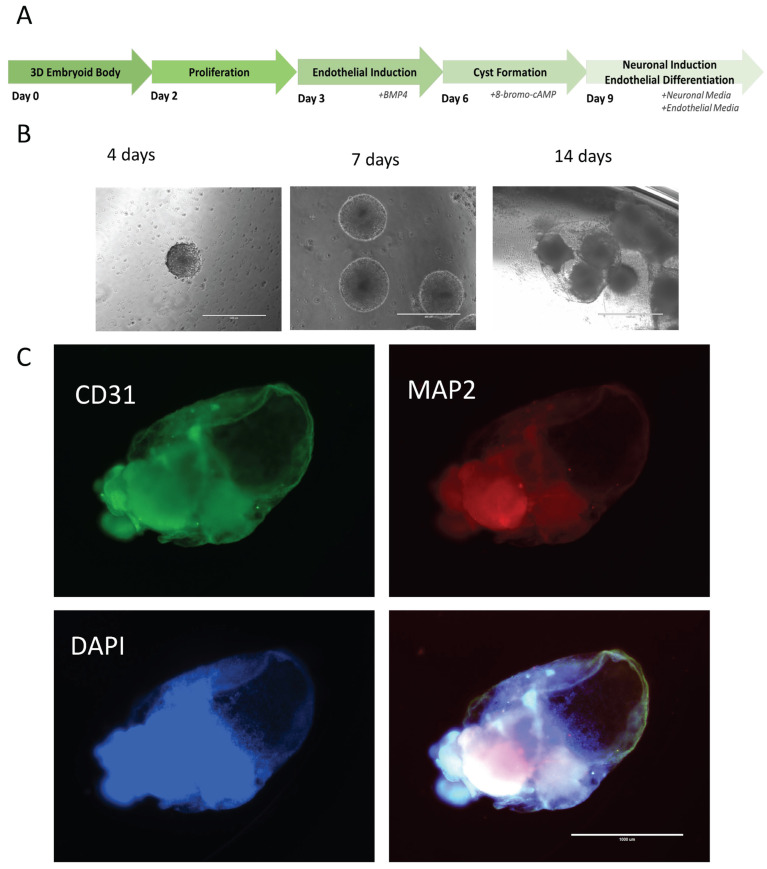
Derive 3D neural organoids with endothelial cells. The 3D organoids were derived from iPSCs following the schematic procedures to achieve vascular endothelial cells and neural cells simultaneously (**A**). The morphological changes during organoid development (**B**). The resulting organoids at the age of 8 weeks were immunostained and showed CD31+ endothelial cells lining a mass enriched with MAP2+ neurons (**C**). Representative images from three independent experiments were shown.

**Figure 2:**
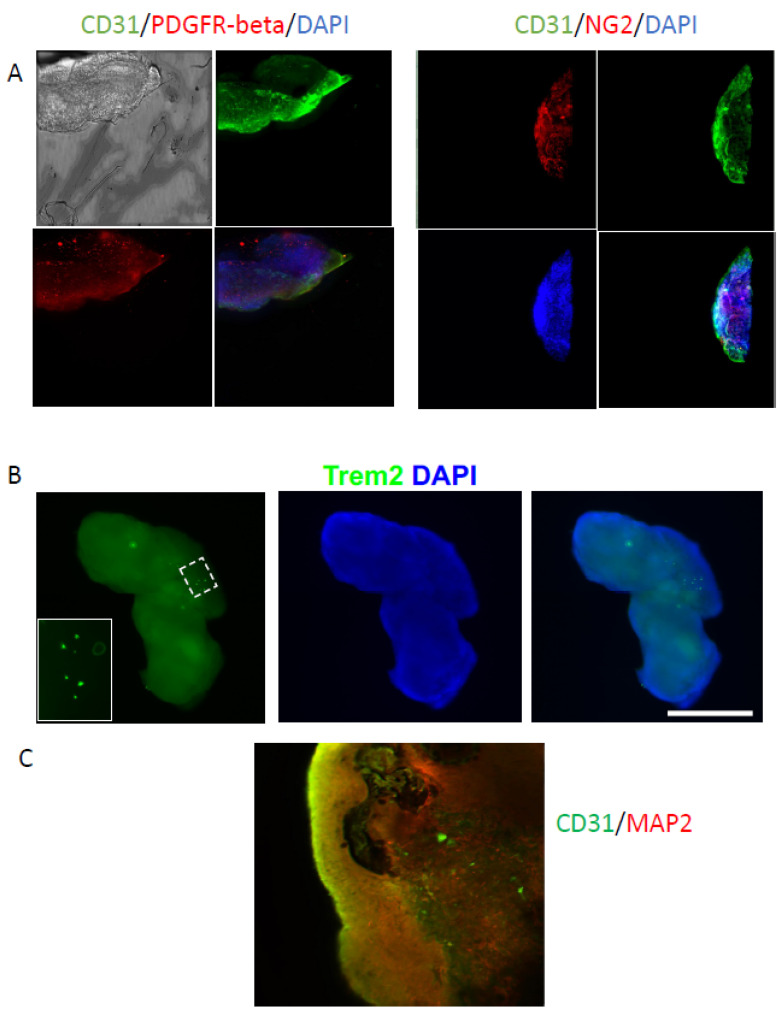
Expression of cell type-specific markers in the organoids. (**A**) After 10 days of neuronal differentiation, pericyte markers PDGFR-beta (red) and NG2 (red) were expressed along with vascular endothelial marker CD31 (green). (**B**) Microglial marker Trem2 (green) was expressed in the organoid. (**C**) Vascular-like structures developed in later mature stage of organoids that immunostained with endothelial marker CD31 (green) and neuronal marker MAP2 (red). Representative images from three independent biological replicates were shown.

To generate a neural organoid similar to the human brain, which consists of neurons, glia and endothelial cells, we developed a protocol to first differentiate iPSC-derived embryoid bodies partially into endothelial cells, then followed by incubation in medium supporting both endothelial and neural cells. By using AggreWells, the uniform-sized embryoid bodies were generated from iPSCs. After 48 h, Activin-A was applied to the medium following Matrigel coating of the embryoid bodies. Then, 24 h later, the media was replaced with DMEM/F12 media containing BMP4 and bFGF to initiate the vascular endothelial cell differentiation. At day 6, the media containing cAMP and VEGF was used to finalize the endothelial differentiation and initiate the neuronal differentiation. After 72 h, the resulting organoids were further cultured in media containing N2 and B27 supplements and VEGF to support both neural and endothelial cells (Figure 1A). Shortly after this stage, the organoids showed a cyst-like structure, consisting of a tightly connected monolayer of cells, and these cells then developed into CD31+ endothelial cells (Figure 1B). A mass core then appeared within the organoids, where most MAP2+ neurons were located. As seen in Figure 1C, after clearance and immunostaining, at 8 weeks after differentiation, the organoids contained both MAP2+ neuronal cells and CD31+ endothelial cells. At this stage, the endothelial cells were mainly lining the organoids while neuronal cells occupied the inner mass. When further cultured until 12 weeks, the CD31+ endothelial cells were seen within the organoids, showing blood vessel-like structures (supplemental Z-scan movie and Figure 2C). To further support that vascularization existence in the organoids, pericyte markers PDGFR-beta and NG2 were coimmunostained with CD31. Both pericyte markers showed certain degrees of colocalization with CD31 (Figure 2A). The resulting organoids also contained a few microglial cells, as shown by the TREM2+ staining cells in the organoids (Figure 2B).

### 3.2. ScRNA-Seq Analysis Showed That EC-Neural Organoids Contain Neurons and Glia Like in Human Brains

**Figure 3:**
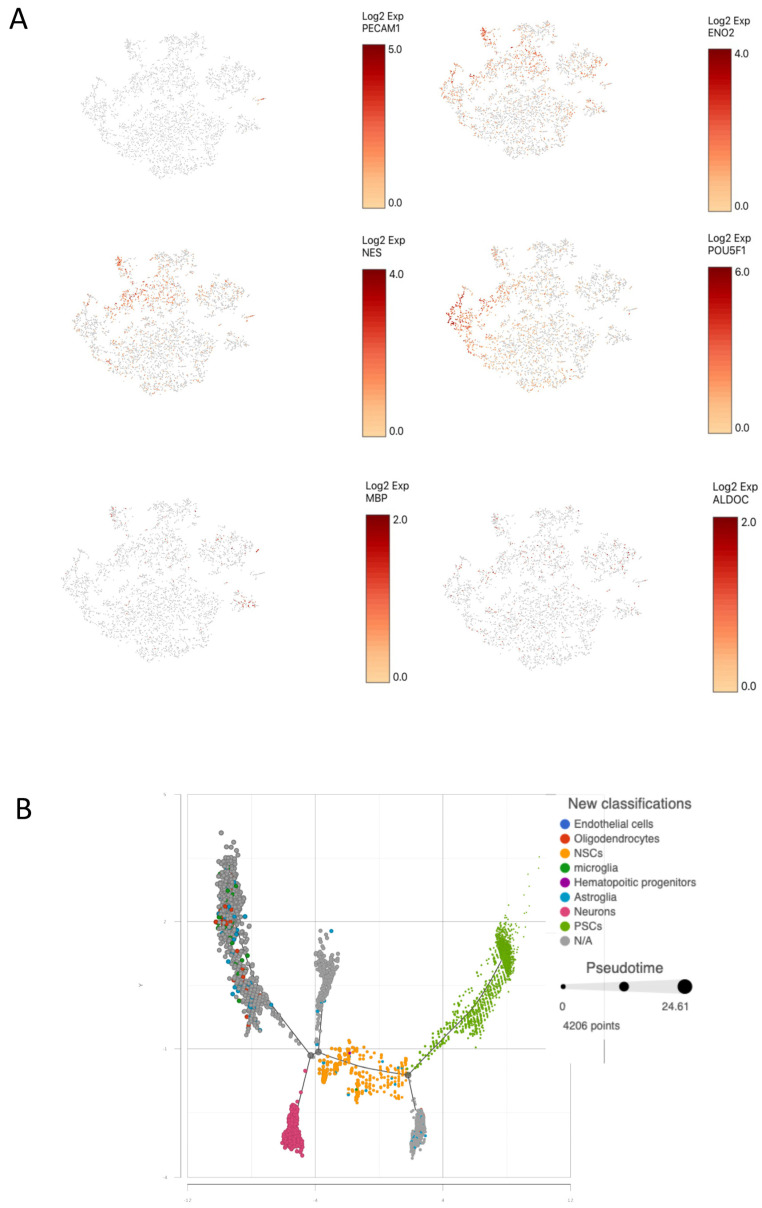
ScRNA-seq analysis of the cellular development of EC-neural organoids. (**A**) The transcripts of cell type-specific markers in four EC-neural organoids were studied using scRNA-seq analysis and t-SNE plots showing the organoids consisted of cells expressing cell type-specific markers such as for endothelial cells (PECAM1), neurons (ENO2), neural stem cells (NES), pluripotent stem cells (POU5F1), oligodendrocytes (MBP) and glial (ALDOC) cells. (**B**) A pseudotime graph showing the trajectory development of the cell types within the organoids.

**Figure 4:**
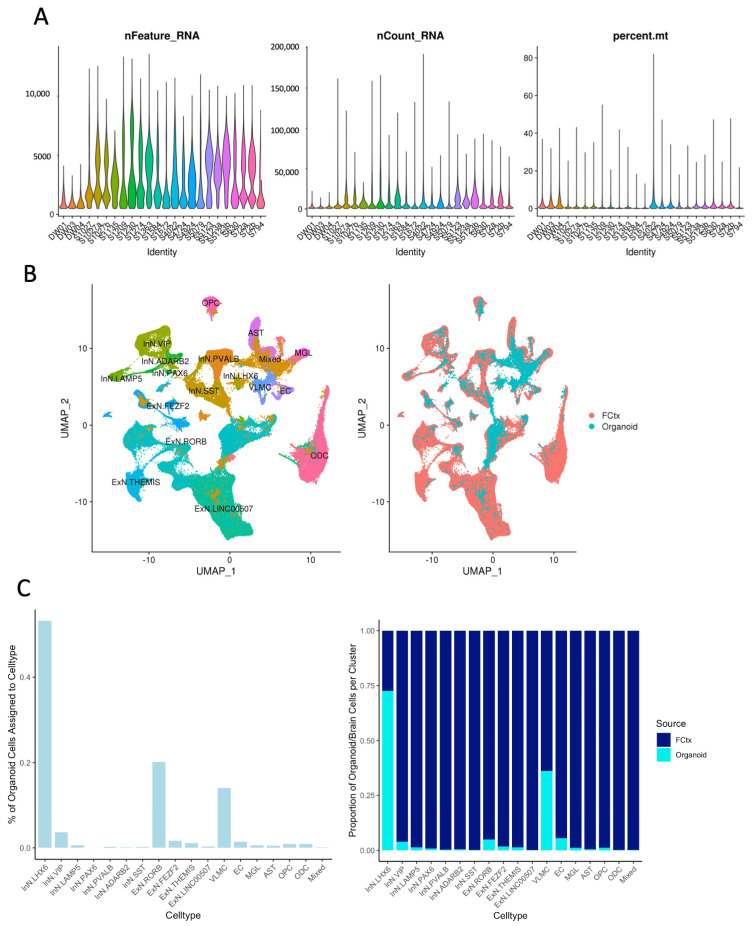
Comparison of cell types within the organoids and human brains. Three scRNA-Seq datasets from the EC-neural organoids (organoid, DW*) and 21 snRNA-Seq datasets from human frontal cortex (Fctx, S*) were compared using methods described in the Methods Section. (**A**) QC plots for single-cell data analysis. Results were presented using UMAP (**B**) and quantified according to specific cell types (**C**).

Four organoids were dissociated separately for scRNA-Seq analysis. The results showed that, as expected, they were built with neurons, oligodendrocytes, microglia, astroglia and a decent amount of neural stem cells and pluripotent stem cells (Figure 3A). A pseudo-developmental trajectory (Figure 3B) showed how these cells were differentiated from pluripotent stem cells over time. When comparing the organoids with the data from human brain samples obtained from the Allen Brain Atlas, the organoids contained varieties of neurons and glia comparable to the types found in human brains (Figure 4).

### 3.3. Gene Profiling in EC-Neural Organoids Emphasized Functions of Angiogenesis and Vascularization

**Figure 5:**
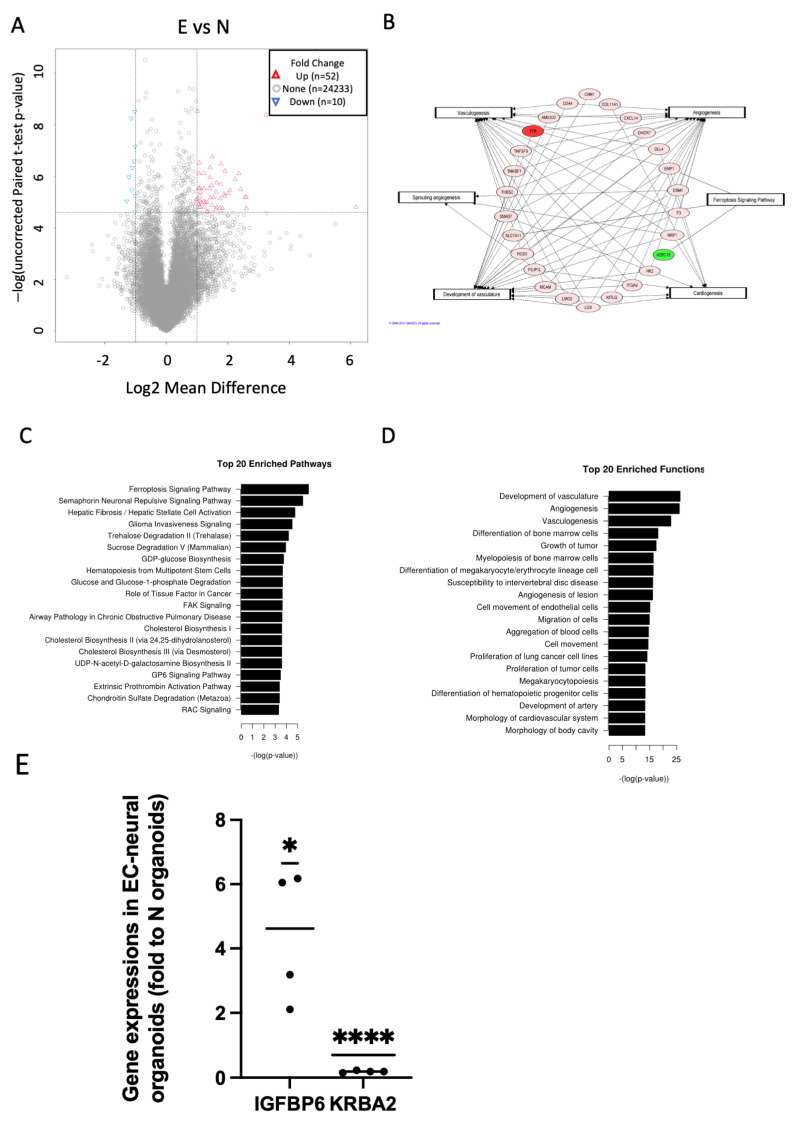
RNA-Seq analysis revealed the difference between EC-neural organoids and cerebral organoids. At 4 weeks old, EC-neural organoids (E) and conventional cerebral organoids (N) generated from 6 iPSC lines were collected for RNA-Seq analysis. Differences in gene expression were observed (**A**), and their relations were plotted as in (**B**). The top 20 enriched pathways (**C**) and functions (**D**) were presented. (**E**) The difference in gene expression of IGFBP6 and KRBA2 between E and N was also confirmed using RT-PCR from four biological replicates. *N* = 4, * *p* < 0.05. **** *p* < 0.0001.

To compare the difference between EC-neural organoids and the conventional brain organoids, which lack endothelial cells, we derived both EC-neural organoids and conventional cerebral organoids from six iPSC lines side by side. At four weeks old, the organoids were collected for gene expression profiling using RNA-Seq analysis. To test for and select differentially expressed genes, the raw expression per gene was first pedestalized in scaled TPM units by adding a value of 2 before undergoing the Log2 transform. Then the cyclic lowess normalization was applied to correct for differences in expression distribution spread and location. Afterward, the mean expression was modeled by the coefficient of variation to identify at what expression value the coefficient of variation exceeded 20% = value = 5. The gene expression less than this value was then filter removed and any values less than this value were floored to this value. Genes were selected to be differential between N and E if they had an absolute linear fold change ≥ 2X and an uncorrected *p*-value < 0.01 by the paired t-test; this provided for a set of 62 genes. As summarized in the volcano plot (Figure 5A). The covariance-based PCA is regenerated using only the differential genes selected and the companion clustered heatmap (Figure S2A,B). In addition, WGCNA (=Whole-Genome Correlation Network Analysis) was also performed in an attempt to find modules of genes that have expression that both covaries with one another and also has correlation with the class of interest = N vs. E. The result is shown in the summary heat map that describes the modules identified (*y*-axis) and their correlation (*p*-value) to N vs. E (Figure S2C). While there are no modules that have a *p*-value < 0.05, the closest two modules that come close are the turquoise module and the gray module. While genes in the turquoise module have lower expression in N than E, the genes in the gray module have higher expression in N than E. The top expressed gene in the turquoise module was IGFBP6, and in the gray module, it was KRBA2, as shown in Figure S2D. The significant differential gene expressions were also confirmed by RT-PCR analysis (Figure 5E). The top 20 enriched pathways and functions were illustrated in Figure 5C,D.

Those differential genes between the E vs. N condition that have connectivity with the top enriched pathway (Ferroptosis Signaling Pathway) and the top enriched functions (Development of Vasculature, Angiogenesis, Vasculogenesis) as well as the other genesis-related functions were plotted in networks as in Figure 5B. The nodes are colored by linear fold change (green = down-regulated, red = up-regulated). Given that all but one are up-regulated, it would seem the E condition would, in turn, be a more “pro” outcome than the N condition per Development of Vasculature, Angiogenesis, and Vasculogenesis.

### 3.4. KRBA2 Is a Critical Factor Regulating Neuronal Differentiation

**Figure 6:**
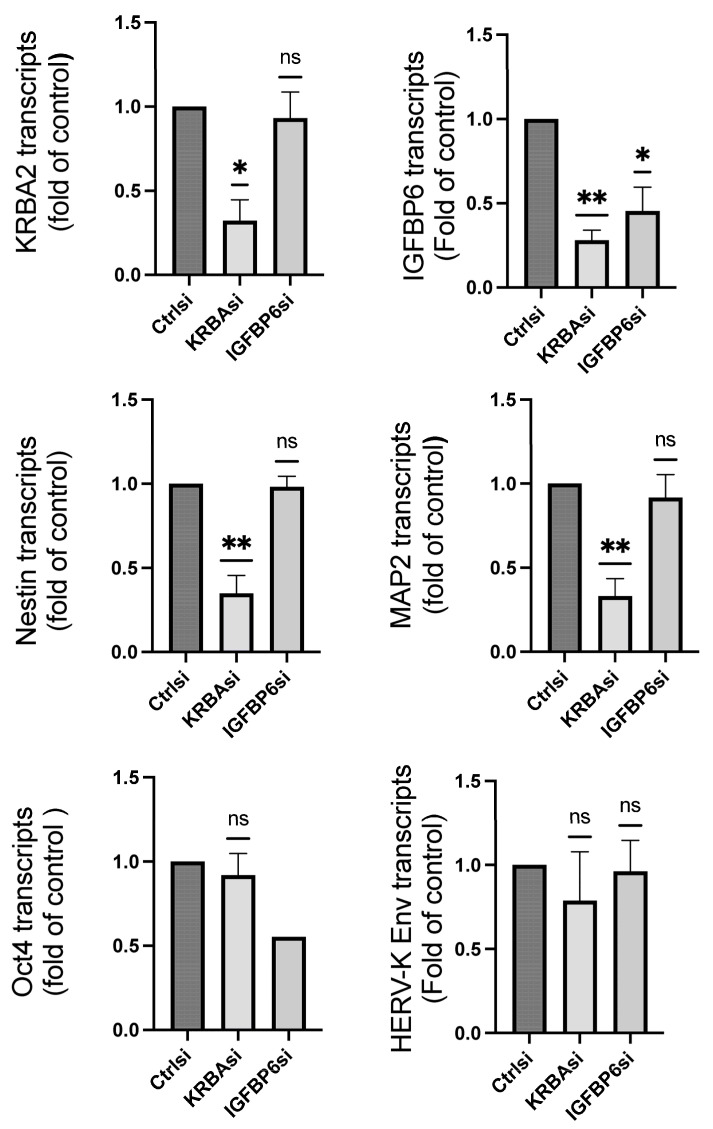
KRBA2 knockout decreased the expression of markers for neuronal differentiation. Neural stem cells were transfected with siRNAs targeting KRBA2 or IGFBP6. The total RNAs were extracted after 48 h for RT-PCR study. The knockout of KRBA2 significantly inhibited gene expression of neuronal differentiation markers Nestin, MAP2 and IGFBP6 but not markers for stemness OCT4 and HERV-K Env. Results were from four biological replicates. *N* = 4. * *p* < 0.05, ** *p* < 0.01, ns, not significant, compared with negative control siRNA.

As expressions of IGFBP6 and KRBA2 showed significant differences between EC-neural organoids and neuronal-only organoids, we used RNAi treatment to knock down the genes in neural stem cells and determined their effects on a variety of genes involved in neuronal differentiation. Although both RNAi treatments knocked down the corresponding genes significantly, the IGFBP6 inhibition showed no significant effect on the genes we checked in the neural stem cells. On the contrary, the inhibition of KRBA2 resulted in significantly lower expression of nestin and MAP2, markers for neuronal differentiation, but no significant effect on OCT4 and HERV-K Env, genes associated with pluripotent stemness (Figure 6). These results indicate that the EC-neural organoids could be used as a tool to study pathways for neuronal specific developments.

### 3.5. EC-Neural Organoids Showed Low Level of ACE2 and TMPRSS2 Expressions Comparable to Published Databases for SARS-CoV-2 Receptors

We took advantage of published human brain databases to determine the expression of certain factors that play important roles in mediating SARS-CoV-2 infectivity. Using Allen’s Atlas microarray database [23] (http://human.brain-map.org (accessed on 18 July 2010), it was found that expression of ACE2 and TMPRSS2 was low across the different brain regions, while FURIN and BSG expression were relatively higher (Figure S5). To determine the possible cell types that may be infected by SARS-CoV-2, we used the cell transcriptomic RNA-Seq data tool in the Allen brain database [23], which confirmed that there was no significant expression of ACE2 or TMPRSS2 in neural cells (Figure S5) (http://celltypes.brain-map.org/rnaseq/human_m1_10x, accessed on 18 July 2010). Similar results were also found in another adult human brain RNA-Seq dataset [26] http://www.gbmseq.org (accessed on 30 October 2020) (Figure S6).

We checked the gene expression of SARS-CoV-2 receptors in the EC-neural organoids. Results showed that ACE2 or TMPRSS2 were expressed in a few cells in the organoids. However, there were more cells with alternative SARS-CoV-2 binding proteins Furin, NRP1 and BSG. (Figure S1). Both glia and neurons express ACE2 and TMPRSS2, though the percentage of positive cells was relatively higher in glial cells compared to neurons (Figure S4). These results agree with the results we observed using online scRNA-Seq databases on the human brain.

## 4. Discussion

The generation of blood vascular endothelial cells has been challenging in human iPSC-derived neural organoids. This is because most neural cells originate from ectoderm, but endothelial cells are from mesoderm. The traditional protocols for making neural organoids involve neural induction as soon as embryoid bodies are formed, which determines the ectoderm fate of the organoids and makes it almost impossible to differentiate into cell types from other germ layers. To introduce blood vessels or vascular endothelial cells into the brain organoids, different approaches have been reported, such as transplanting human neural organoids into rodent brain, mixing the differentiated HPCs or mature microglia into neural organoids and combining differentiated blood vascular organoids with neural organoids to make assembloids. However, normal human brain development is a process involving constant interactions between neural development and blood vascular development. To incorporate separately differentiated cells risks missing these early interaction aspects. By serially inducing endothelial and neural differentiation on iPSC-derived embryoid bodies, we report here for the first time having generated 3D organoids consisting of both neural and inherent endogenous vascular endothelial-like cells.

During human brain development, blood vascular development consists of the initial vasculogenesis and then angiogenesis. Vasculogenesis is when the angioblasts differentiate into endothelial cells and give rise to the de novo vascular networks [27]. The necessity of human brain vascular system development begins when the neural tube is isolated from the surrounding amniotic fluid by dense connective tissue and the meninx primitiva. From the later, the primitive vascular loops develop and form the “meningeal meshwork”, within which preferential streams appear and give rise to the subarachnoidal brain arteries [28]. The typical brain angiogenesis thus is characterized by proliferation of endothelial cells which penetrate the neural tissue from the brain surface to form interconnected channels. Based on this knowledge, we mimic the in vivo human brain angiogenesis by starting endothelial cell induction first. Endothelial induction using BMP-4, bFGF, plus Matrigel treatment results in a relatively enclosed environment for organoid development when the outlayer PECAM-1-positive endothelial cells proliferate, preferably on the Matrigel coating layer, and make a cyst. The cyst prevents the inner cells from fully interacting with the outside endothelial-inducing factors, allowing them to differentiate into other cell types such as neural cells. It is reported that endothelial cells with stem cell properties exist as CD157 (also known as Bst1) positive cells. The CD157+ endothelial cells contribute to endothelial cell turnover and are capable of reconstituting hierarchical blood vessel networks in vivo [29]. We observed abundant CD157+ cells in the organoids using scRNA-Seq analysis, indicating there are endothelial stem cells and the potential of the organoids to maintain their vascular system. The vascular endothelial-like cells would only penetrate the neural tissue at later time points when the organoids reached ages beyond 8–12 weeks. We also observed connective tissue markers within a substantial number of cells in the organoids. These results indicate that in the organoids, the endothelial cells and vascular-like structures develop in a similar way as in the human brain, by isolating the neural tissue with connective tissue and a meningeal surface first, and then penetrating from the surface into the neural tissue mass. As a result, the vascular-like structures in the organoids only happen at a later time as well. It is reasonable to believe that the angiogenesis within the organoids is also a dynamic process that would continue to develop with the organoid age.

However, we should point out that the chances of tissues from other germ layers/organs may also rise within the organoids, likely due to the early enclosure of the cell mass from the immediate environment, which prevents direct perfusions and subdues the effect of directional differentiation. In fact, we have observed AFP (alpha-fetoprotein) in the early organoids, which then decreases over time, likely because of neuronal-selective media. We also observed other non-specific markers, including a substantial number of pluripotent cells in the organoids using the RNA-Seq analysis. This delayed differentiation of pluripotent stem cells may be due to the same isolation effect, and the pluripotent status is likely transitional.

To explore the potential of the organoids in neuronal development studies, we compared the gene expression profiles between 3D organoids comprising endothelial cells and without endothelial cells. The top differential pathways are related to endothelial cells and blood vessel differentiation and development. IGFBP6 expression is significantly higher in the ones with endothelial cells, while KRBA2 is higher in organoids without endothelial cells. IGFBP6 expression has been reported in endothelial cells [30] and plays important roles in blood vascular development, including the maintenance of endothelial integrity [31] and vascular smooth muscle cell proliferation and morphology [32]. It has been reported that serum IGFBP6 level is higher in young mice than in aged ones [33]. IGFBP6 is secreted by various stem cells and blasts together with TIMP2 [34,35,36], a factor playing a key role in revitalizing aging hippocampal function [37]. Published reports suggest that IGFBP6 may play some roles in the process of preserving cells against aging effects, such as resisting cell senescence [38] and alleviating neurodegenerative disorders including PD [39]. Thus, endothelial cells, by secreting IGFBPs, could help reconstitute brain hemopoiesis. We found upregulated IGFBP6 expression in EC-organoids compared to neural-only organoids, suggesting IGFBP6 could play a role in brain development and function, which could not be observed in a traditional brain organoid alone.

We found there was a higher level of KRBA2 expression in the neural-only organoids. When KRBA2 expression was knocked down by siRNA in neural stem cells, we observed a decrease in several neural-specific gene expressions, but not in pluripotent stemness gene OCT3/4, indicating that KRBA2 may play a role in regulating neural differentiation. KRBA2 was thought to be a result of horizontal transfer of the GINGER2 transposon from insect to the human genome. The function of KRBA2 is unclear, although a similar KRAB domain has been reported to play a repressive role in gene regulation by recruiting transcriptive repressor complexes [40]. It has been suggested that KRBA2 may be adopted by hosts as a defense against other mobile elements. Given that retroelement activities are usually higher in stem cells but decrease during differentiation, KRBA2 may indeed function as a transcriptional factor facilitating neural differentiation given its broad band enhancing effect on neuronal specific gene expressions. The role of KRBA2 in regulating neural development warrants further investigation.

The COVID-19 pandemic has had an unprecedented impact on human life. While neurological manifestations are commonly seen in patients with the infection, they can also be the presenting manifestation in some [41]. In rare cases, SARS-CoV-2 has been demonstrated in the cerebrospinal fluid in patients with meningo-encephalitis [42,43] and in the brain at autopsy in endothelial cells by electron microscopy [44,45]. In another patient, the virus was demonstrated budding from myelin sheaths in the medulla and olfactory pathways [46]. Allen Atlas’s microarray database shows that both ACE2 and TMPRSS2 expression are low across brain regions, while the expression of FURIN and BSG is relatively higher. The scRNA-Seq data in the Allen Brain database confirms there is no significant expression of ACE2 or TMPRSS2 in neural cells. The lack of TMPRSS2 expression and low level of ACE2 expression in neural cells are consistent with another brain scRNA-Seq dataset from adult brain samples from UCSF. A recent study used 3D brain organoids to study the infectivity of SARS-CoV-2 in the brain and found that the virus was capable of infecting the organoids [47]. However, this model of brain organoids lacked endothelial cells or a blood–brain barrier (BBB. Vascular endothelium has been reported to be the primary target of SARS-CoV-2 [44] and a high level of ACE2 is expressed in brain vascular pericytes in a mouse brain [48], indicating an important role in neuropathogenesis of the infection. On the other hand, Furin, a proprotein convertase, could pre-activate the SARS-CoV-2 spike protein, and thus reduce its dependence on the target cell proteases for cell entry [49]. We examined the SARS-CoV-2 receptors on the EC-neural organoids. Our results indicate that ACE2 or TMPRSS2 were expressed in a few cells in the organoids. However, there were more cells with alternative SARS-CoV-2 binding proteins: Furin, NRP1 and BSG. (Figure S1). Both glia and neurons express ACE2 and TMPRSS2, though the percentage of positive cells was relatively higher in glial cells compared to neurons (Figure S4). These results agree with the findings in the human brain. Thus, the 3D EC-neural organoids can potentially be used to study neuroinfectious disorders such as SARS-CoV-2.

The EC-neural organoids contain inherent endothelial cells, initially covering the organoids on the surface and later penetrating the mass to make vascular-like structures. It is highly consistent with the process of human embryonic brain development, when the perineural vascular plexus covers the developing neural tube into which vessels subsequently penetrate [50]. The initial membrane and the later more vascular-like structures could separate the neural cells from the external environment and more closely resemble in vivo conditions, although the vessels lack blood circulation. Whether the vascular-like endothelial cells make a BBB and their functions are still unknown and further characterization is needed. At least, the EC-neural organoids provide a model suitable for studying neural infectious disorders where endothelial cells are targets or used to mediate neural damage.

As a summary, by serial induction of differentiation, we make a 3D organoid containing vascular endothelial and neuronal cells, mimicking a vascularized developing brain. These 3D human organoids could be a useful tool to study human development involving endothelial and neuronal cell interactions and for neural infectious disorders when the endothelial cells are pathologically targeted.

## Data Availability

All relevant data supporting the key findings of this study are available within the article and its Supplementary Information files. The RNA-Seq data are deposited in the public domain: https://www.ncbi.nlm.nih.gov/geo/query/acc.cgi?acc=GSE330092, accessed on 15 June 2026. Software used in this study includes 10X Genomics Loupe Browser 4.0.0 (https://support.10xgenomics.com, accessed on 21 December 2021).

## Supplementary Materials

The following supporting information can be downloaded at: https://www.mdpi.com/article/10.3390/cells15131192/s1, Figure S1: sc-RNA-Seq analysis on EC-neural organoid for SARS-CoV-2 infectivity related proteins; Figure S2: RNA-Seq analysis on differential gene expressions between EC-neural organoids and conventional neural organoids; Figure S3: Immunostaining of the organoids for endothelial cell marker CD31 and neuronal marker beta-III-tubulin; Figure S4: Expressions of SARS-CoV-2 binding associated proteins in different cell types in 3D Endo-neural organoids; Figure S5: scRNA-Seq data from Allen Brain Atlas showing low levels of ACE2 and TMPRSS2 expression across brain cell types; Figure S6: scRNA-Seq data from http://www.gbmseq.org/ showing low levels of ACE2 and TMPRSS2 expression in brain cells; Figure S7: Pearson correlation and UMAP analysis between EC-neural organoids and FCtx; Table S1: Material list; Table S2: Oranoid cells per sample per cell type in broader catagories; Table S3: Person_correlation table: Organoids vs FCtx; Table S4: Intergated data analysis between EC-organoids and FCtx; Video S1: CD31 positive (green) vascular like structures in EC-neural organoid.
