# Supplementary figures and images for: Generation of Blood Vascular Endothelial-Neural 3D Organoids by Serial Induction of Differentiation on Human iPSC-Derived Embryoid Bodies
Source: Cells. 2026 Jun 30;15(13):1192. doi: 10.3390/cells15131192 (PMC13359818; doi:10.3390/cells15131192)

SFig.1

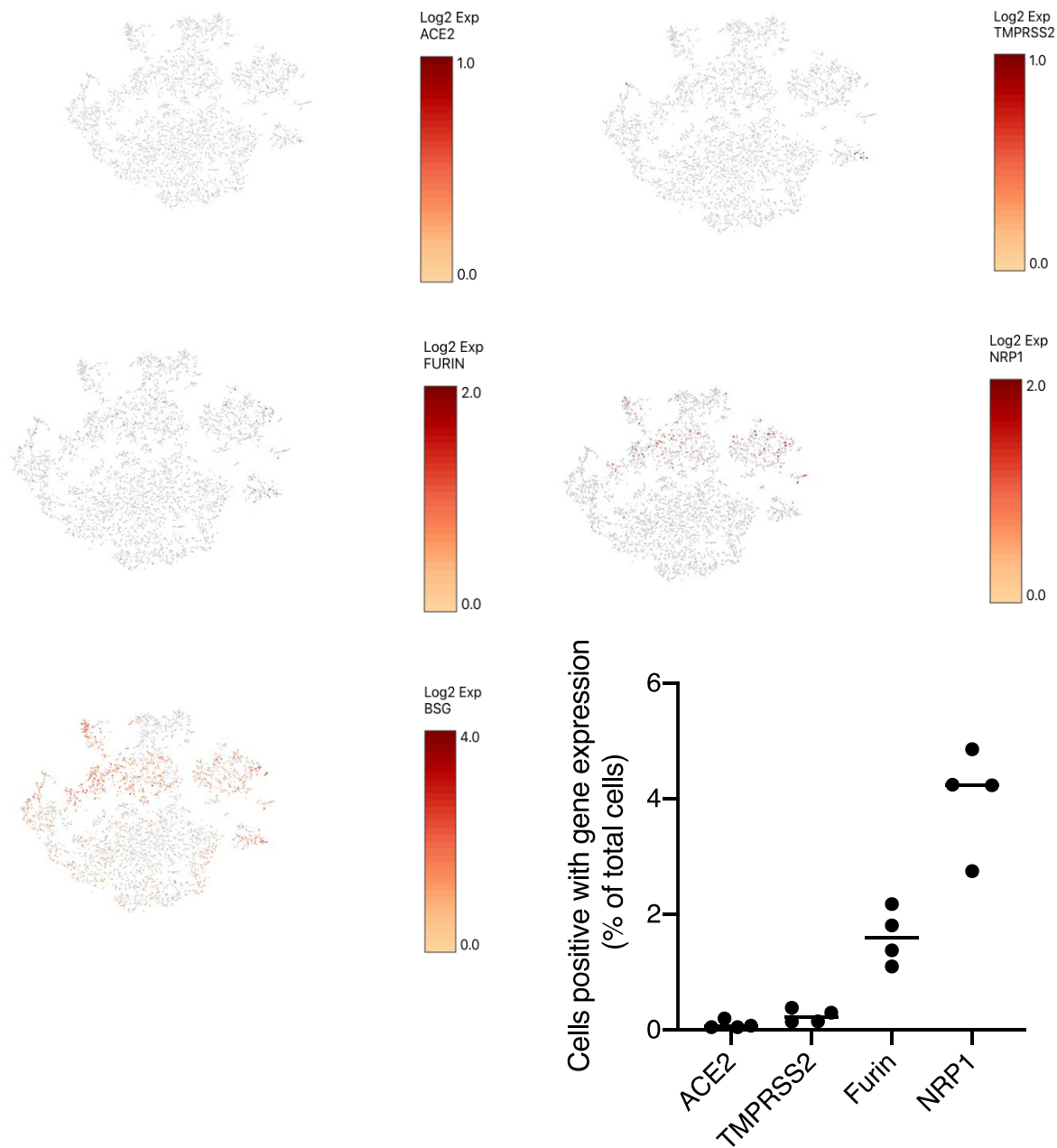

# A B SFig.2

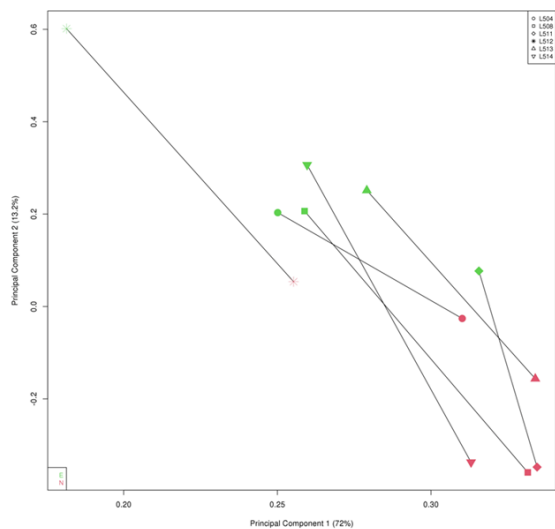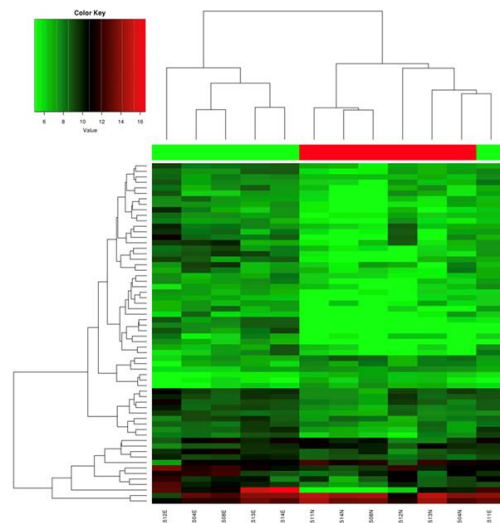

## C

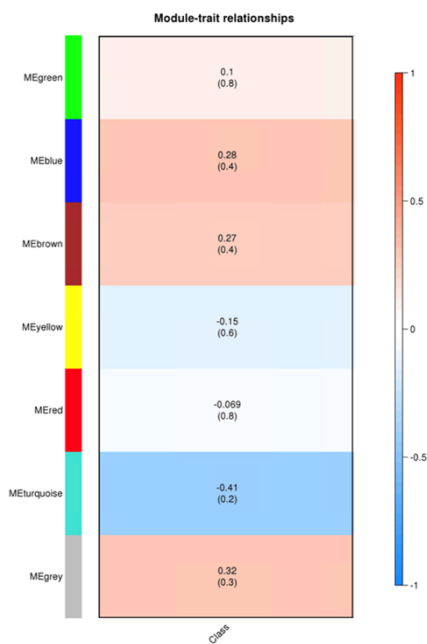

## D

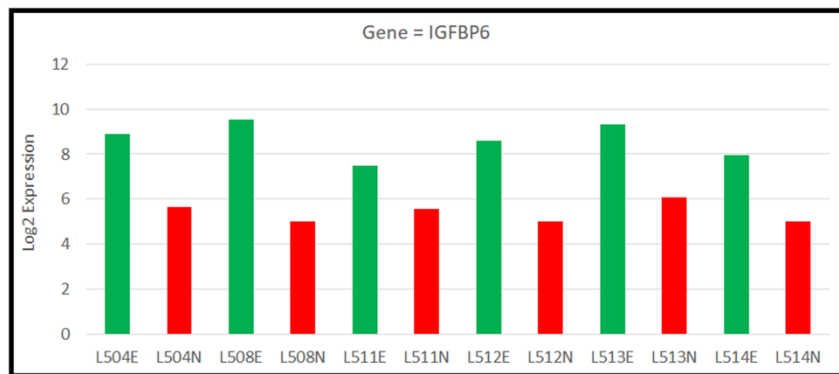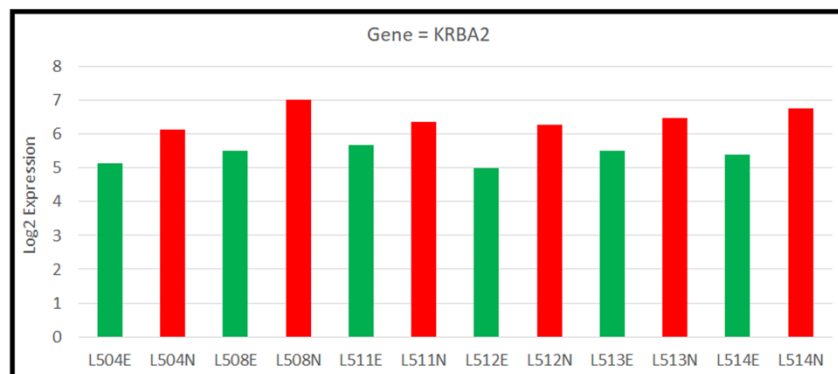

SFig.3

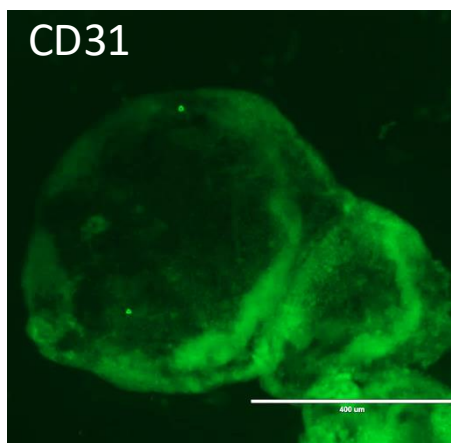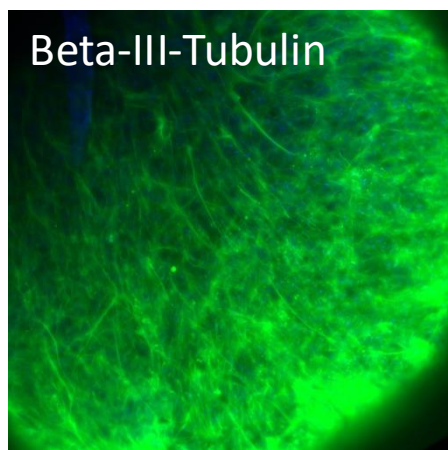

SFig. 4

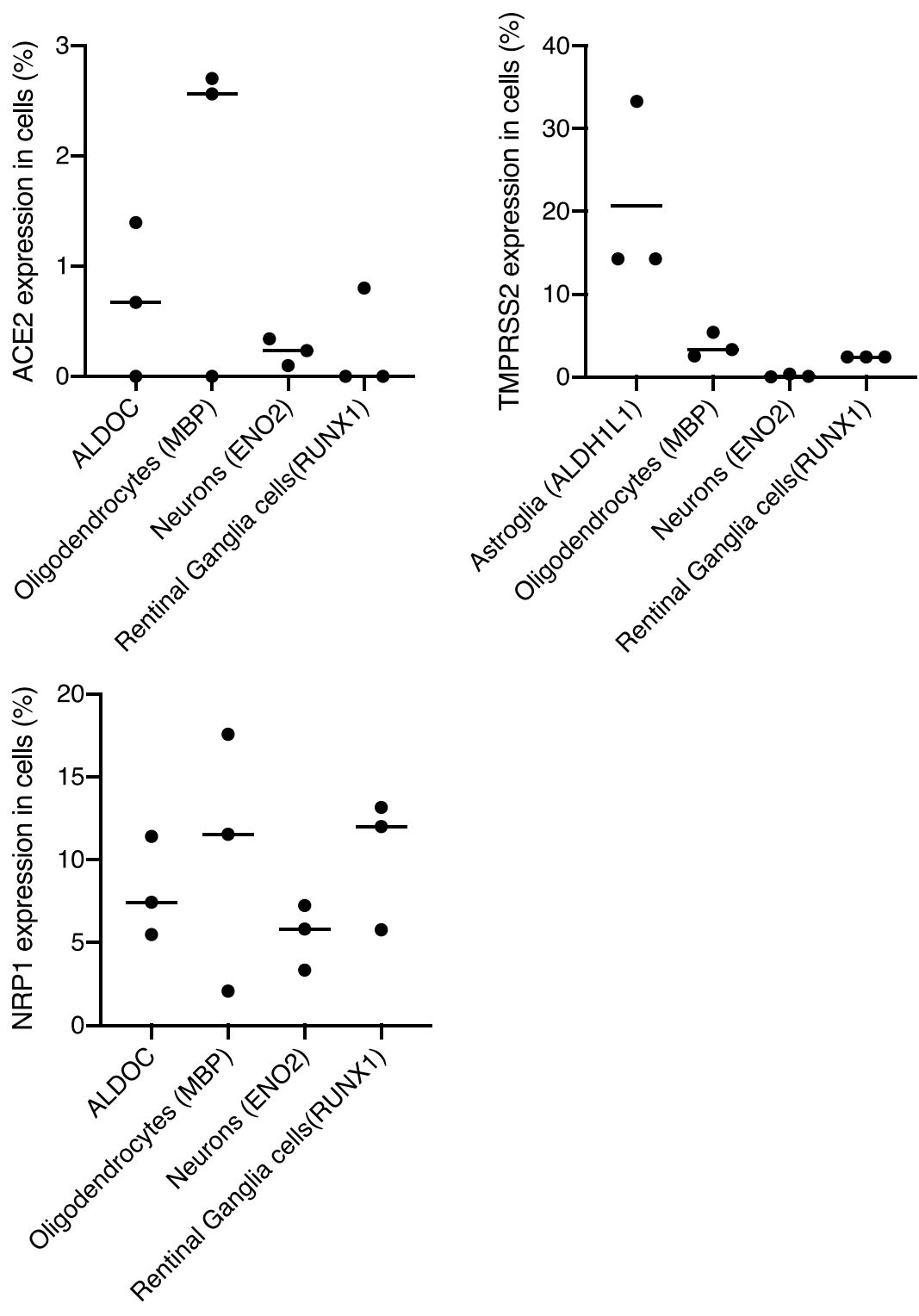

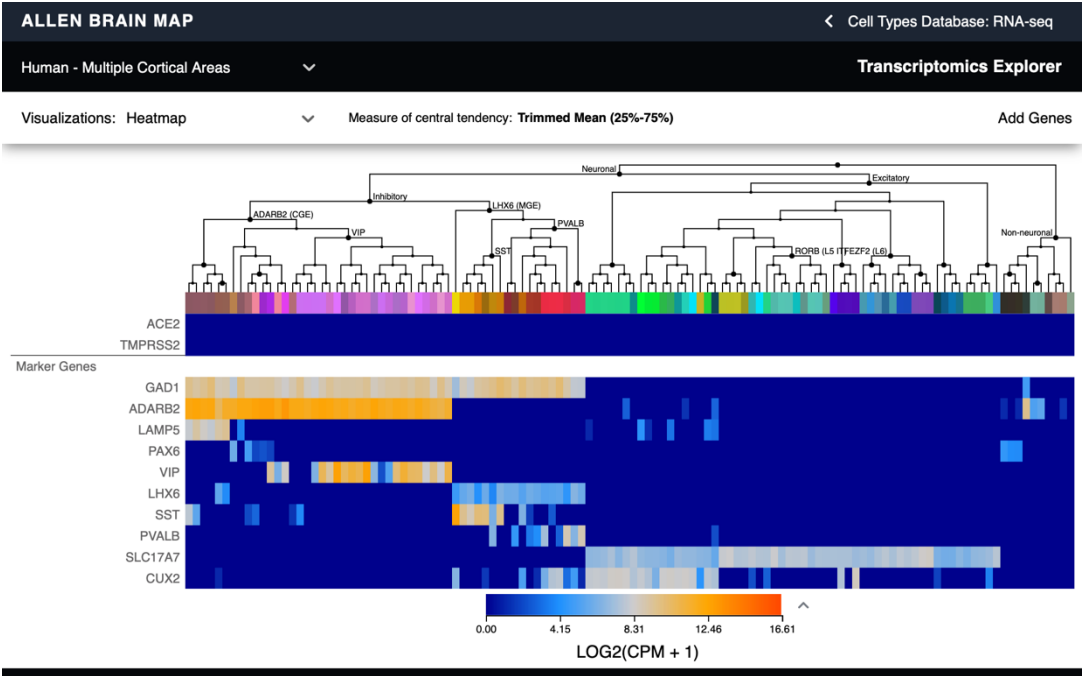

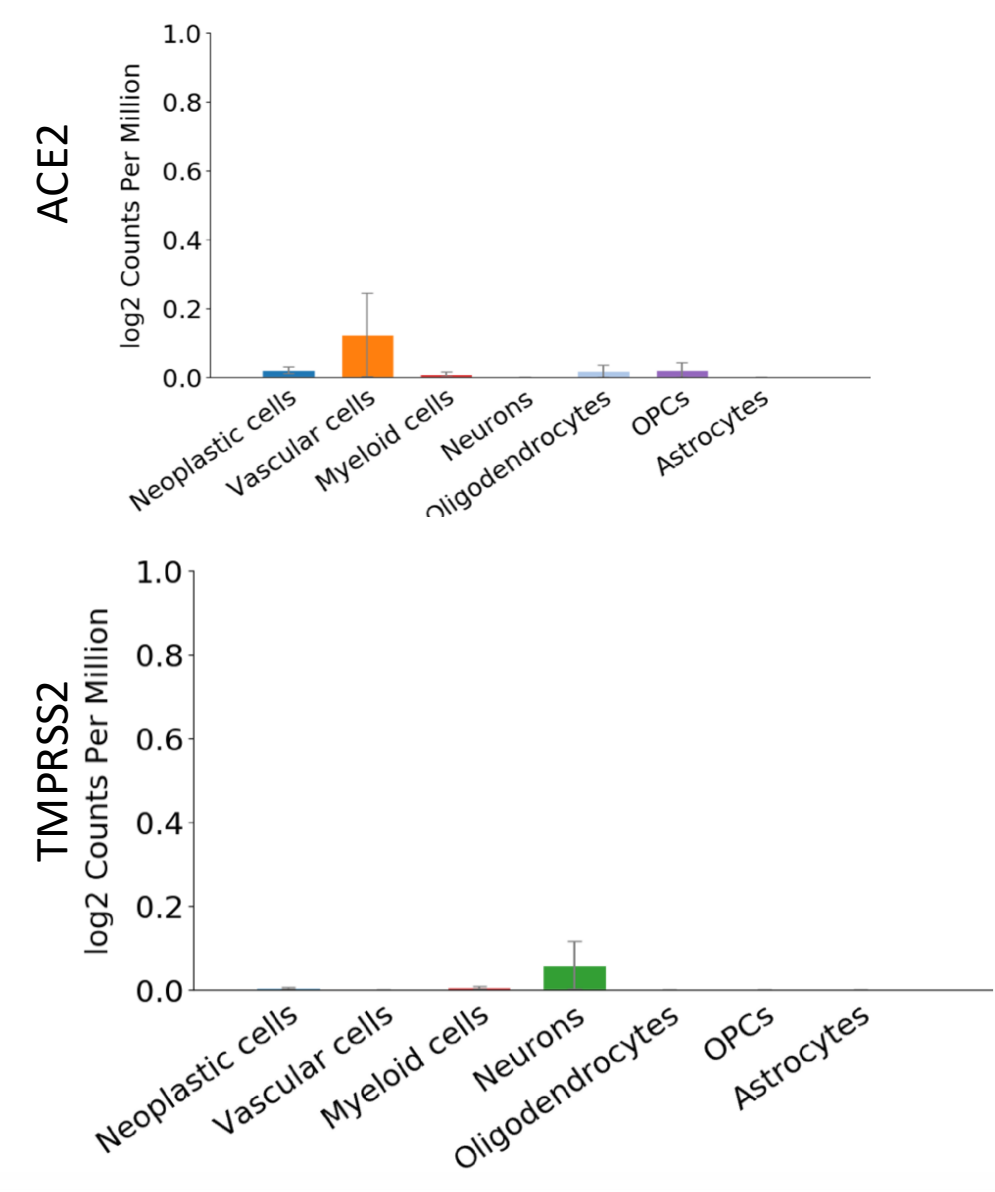

SFig.7

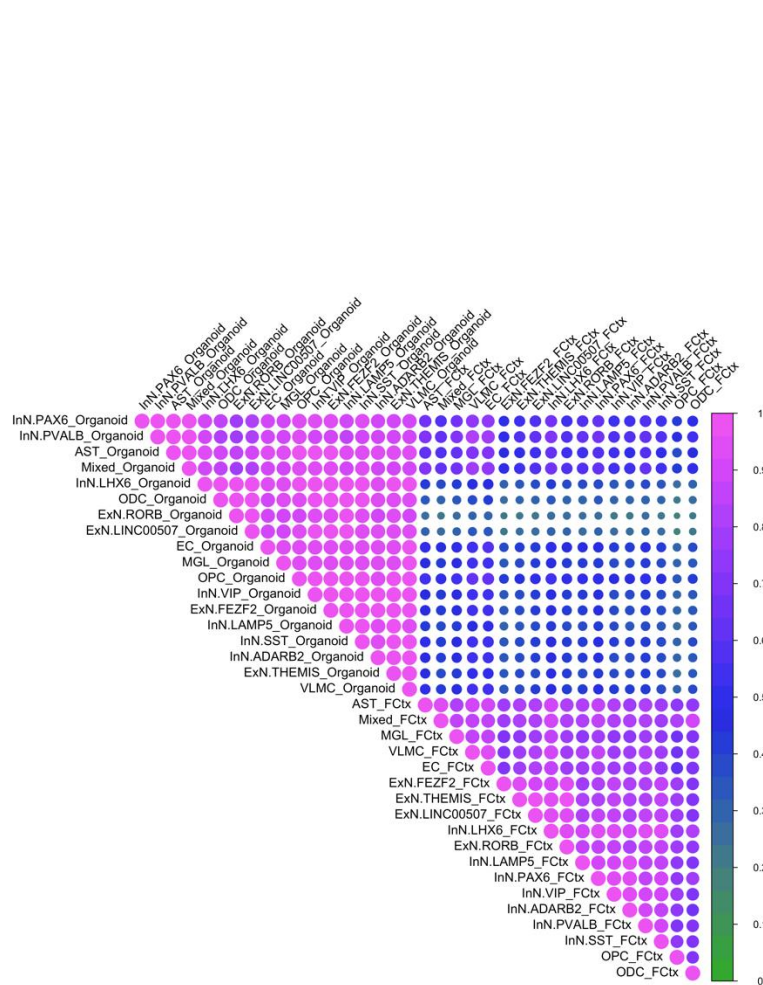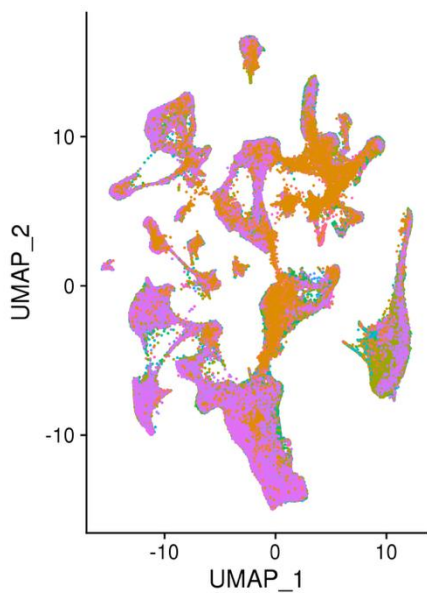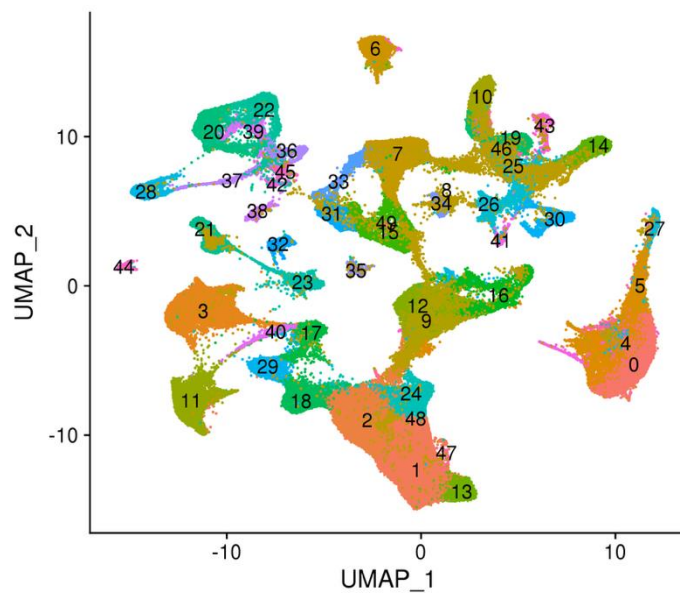

Supplement: Supplementary file 1 [file cells-15-01192-s001.zip › supplemental/SFigs Cells.pdf]
